# Sheehan’s Syndrome in a Patient With Factor XI Deficiency

**DOI:** 10.7759/cureus.62328

**Published:** 2024-06-13

**Authors:** Jayaditya Ghosh, Satyender Dharamdasani, Girish Parthan, Ravi Shah, Pinaki Dutta

**Affiliations:** 1 Endocrinology, Postgraduate Institute of Medical Education and Research, Chandigarh, IND; 2 Hematology, Postgraduate Institute of Medical Education and Research, Chandigarh, IND; 3 Endocrinology, Renai Medicity, Cochin, IND; 4 Endocrinology, Postgraduate Institute of Medical Education and Research, Ahmedabad, IND

**Keywords:** bleeding, coagulation, hypopituitarism, factor xi deficiency, sheehan’s syndrome

## Abstract

Sheehan’s syndrome (SS) is a condition characterized by panhypopituitarism that generally occurs after an episode of postpartum bleeding. There are certain hypotheses regarding the development of SS in the postpartum period. Coagulation factor abnormalities have been reported to be associated with SS. Associated hypothyroidism and hypocortisolism have been found to cause coagulation abnormalities. After the correction of the hypothyroidism and hypocortisolism, there is a gradual correction of the coagulation abnormality. In our case, a middle-aged woman presented with recurrent episodes of hospital admission because of generalized weakness and fever. She was found to have a biochemistry profile suggestive of hypopituitarism with preserved gonadal function. Her hemogram was normal, but the coagulogram showed a prolongation of activated partial thromboplastin time with a near-normal prothrombin time. She was evaluated and found to have factor XI deficiency. In the background of excessive vaginal bleeding and hypopituitarism, a diagnosis of SS was made. The presence of factor XI deficiency may have led to excessive bleeding and the development of SS. To the best of our knowledge, there is no reported association of factor XI deficiency with SS in the literature, and this is the first reported case.

## Introduction

Sheehan’s syndrome (SS) is a state of panhypopituitarism that commonly occurs after an episode of excessive postpartum bleeding. The disease may evolve over a period of several years with a smoldering course and present with subtle clinical features. The exact pathophysiology of SS is unclear, and it is believed that the increase in the size of the pituitary during pregnancy, as well as hypotension or shock during the postpartum period, results in necrosis or ischemic damage to the pituitary in genetically predisposed individuals. Coagulation factor abnormalities have been reported in association with SS in the past, as well as in patients with other causes of hypopituitarism [1]. Here, we report a patient with SS with coagulation factor XI deficiency.

## Case presentation

**Figure 1:**
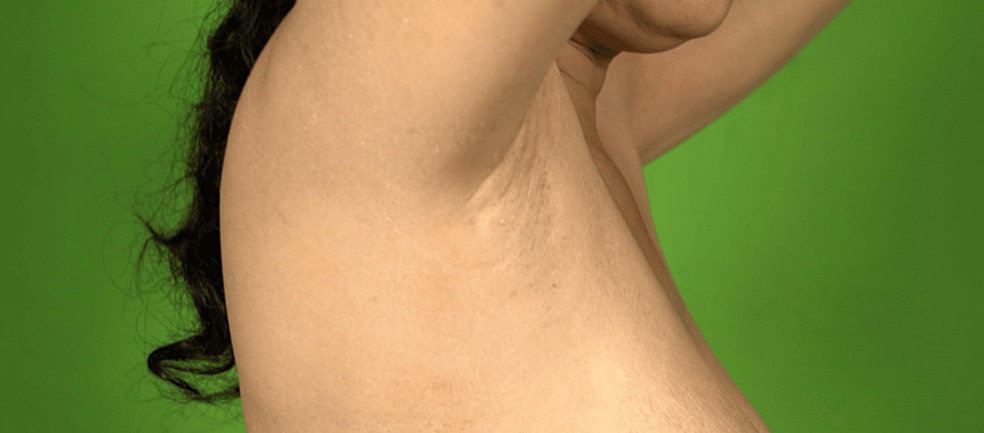
Absent axillary hair

**Figure 2:**
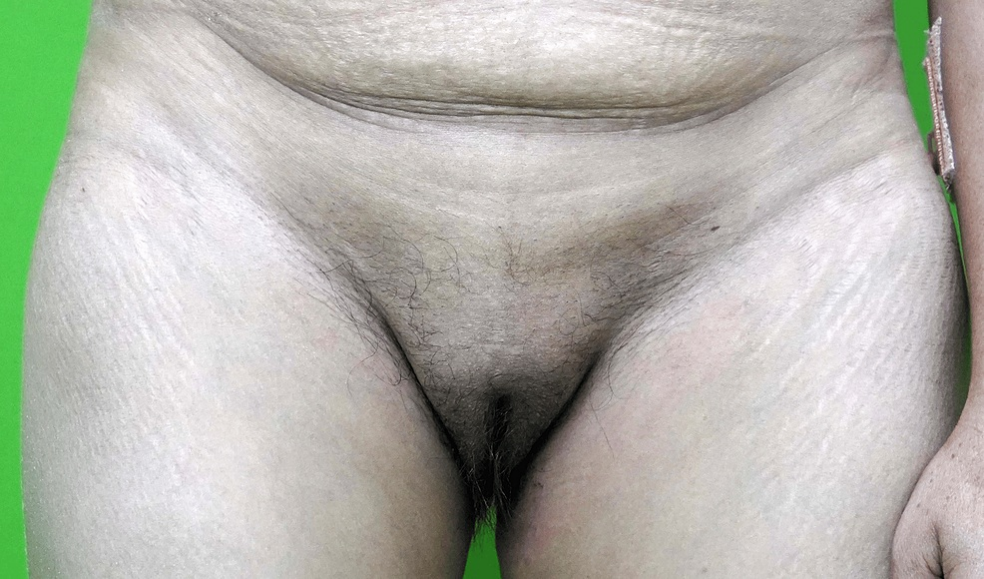
Sparse pubic hair

A 37-year-old woman presented with a history of generalized weakness for the last three to four months. She had extreme fatigue and had a history of cold intolerance. Additionally, she had a history of low-grade fever, ranging around 100 °F, on and off for the same duration. During this time, she encountered multiple episodes of dizziness, vomiting, and low blood pressure (80/60 mmHg), leading to her admission to a local hospital. After receiving IV fluids and nonspecific treatment, she was discharged. For the past nine years, she has been experiencing oligomenorrhea, with amenorrhea occurring over the last five months. She has three children, with her last childbirth being nine years ago. During that delivery, she suffered a severe postpartum hemorrhage, necessitating a blood transfusion. There was no history of bleeding episodes prior to this event or any indications of autoimmunity. Her family history was not significant. Upon evaluation, she appeared pale with a shallow complexion and facial puffiness. Physical examination revealed breast atrophy along with absent axillary hair (Figure 1) and sparse pubic hair (Figure 2).

Her blood pressure was 80/50 mm Hg with no postural drop. Considering her past history of postpartum hemorrhage, a diagnosis of SS is considered. Hormonal evaluation revealed deficiencies of cortisol, thyroid, and growth hormone (Table 1).

**Table 1:** Relevant biochemical investigations for the patient

Parameters	Baseline	After three months	Reference range
Thyroid-stimulating hormone	1.87 uIU/ml	0.0133 uIU/ml	(0.27-4.2)
Total T3	0.43 ng/ml	1.56 ng/ml	(0.8-2)
Total T4	2.7 ug/dl	11 ug/dl	(4.8-2.7)
Thyroid peroxidase	7.20 IU/ml		(<9)
Follicle-stimulating hormone	5.46 mIU/ml	5.12 mIU/ml	(1.5-12.4)
Luteinizing hormone	2.27 mIU/ml	1.56 mIU/ml	(1.7-8.6)
Estrogen	72.7 pg/dl	21 ng/dl	(5-35)
Cortisol	48.5 nmol/L	-	(171-536)
Adrenocorticotropic hormone	7.14 pg/ml	-	(7.2-63.3)
Dehydroepiandrosterone sulfate	0.200 ug/dl	-	(60.9-337)
Growth hormone	0.03 ng/ml	-	(0-2.5)
Insulin-like growth factor 1	19.4 ng/ml	-	(86.5-222)
Prolactin	1.65 ng/ml	-	(4-15.2)
Prothrombin time	18.8 seconds	-	(11-13.5)
Activated partial thromboplastin time	102 seconds	-	(30-40)
Factor IX assay	0%	4.60%	(65-145)
Inhibitor screening	-	Negative	-
Lupus anticoagulant	Negative	Negative	-

**Figure 3:**
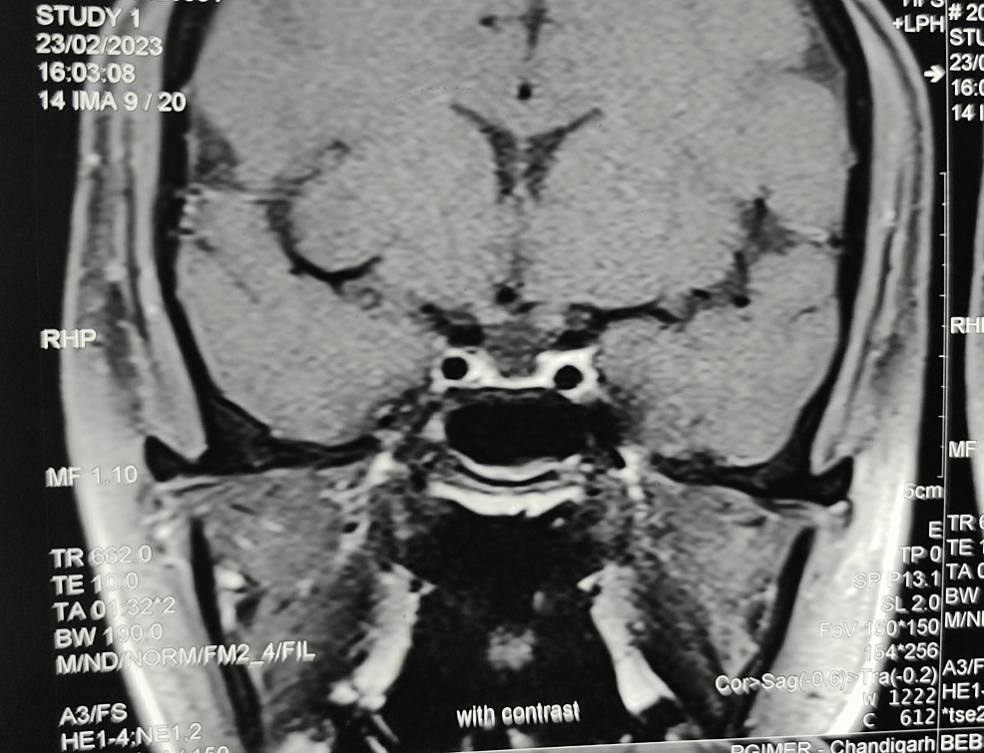
MRI showing an empty sella

An MRI of the sella revealed an empty sella (Figure 3).

The patient was started on thyroid (100 μg) and cortisol (100 mg in divided doses) replacement. On the second day of hospitalization, she had menstrual bleeding and required a change of five to six pads per day. Her hemogram was normal, but the coagulogram showed a prolongation of activated partial thromboplastin time (aPTT) with near-normal prothrombin time (PT). A possibility of anti-phospholipid syndrome or acquired von Willebrand disease was considered in the backdrop of SS. She had an endometrial thickness of 8 mm on ultrasonography, and vaginal bleeding was managed conservatively, which stopped after four days. Her serum estradiol was 72.6 pg/mL, without any history of hormonal supplementation. The anti-phospholipid anticoagulant profile (including anti-cardiolipin and anti-beta2 glycoprotein, IgM and IgG antibodies, and lupus anticoagulant) was negative. The plasma mixing studies showed factor XI deficiency (factor XI levels: 0%, normal levels: 65-145%) with normal levels of other coagulation factors. Even after correction of the thyroid and cortisol deficiencies, her aPTT was prolonged, and factor XI deficiency persisted (factor XI level: 4.6%). So, a diagnosis of SS with preserved gonadal function and congenital factor XI deficiency was made. The inhibitor screen was negative.

During the three-month follow-up, the patient continued hydrocortisone and thyroid hormone supplementation, reporting improved well-being and reduced facial puffiness. She remained on an irregular menstrual cycle due to an intact gonadotropin axis. We report this case as factor XI deficiency has not been reported to be associated with SS.

## Discussion

Our patient had features suggestive of hypothyroidism, hypocortisolism, and empty sella on imaging, with a history of severe postpartum hemorrhage in the past. Laboratory parameters were suggestive of panhypopituitarism, and she was diagnosed with SS. She had excessive vaginal bleeding after starting thyroid and cortisol replacement, which was managed conservatively. An increased aPTT with a near-normal PT, coagulation factor assays, and plasma mixing studies confirmed factor XI deficiency. In our patient, we hypothesize that the underlying factor XI deficiency contributed to the excessive postpartum hemorrhage, which could have resulted in the development of SS. She had two normal vaginal deliveries in the past and did not have any complications.

There are multiple hypotheses regarding the pathogenesis of SS. The most accepted one is the mismatch between blood supply and demand in the immediate postpartum period. An enlarged pituitary during pregnancy, associated vasospasm, small sella turcica, vascular compression, thrombosis, and coagulation abnormalities may also contribute to the pathogenesis. Inherited abnormalities in the coagulation factors have also been reported to be associated with SS [2]. Deficiencies of factors VIII and IX were reported in patients with hypothyroidism [3]. Increased bleeding time, PT, and aPTT, along with deficiencies of factor VIII and von Willebrand factor (vWF), were reported in patients with overt hypothyroidism, which was reversible after thyroid hormone replacement [4]. Two studies have evaluated coagulation profiles in patients with SS. In a study involving 32 women with SS, PT and aPTT were reduced with normal vWF activity and increased fibrinogen and D-dimer [5]. In another study that evaluated platelet function and coagulation profiles in 50 women with SS, PT and international normalized ratio (INR) were prolonged, with normal platelet function, aPTT, and clotting factor levels. However, the effect of hormone replacement on prolonged PT and INR was not evaluated, and the study concludes that patients with SS may be predisposed to bleeding, and coagulation disorders may be the underlying cause of postpartum hemorrhage [6]. Deficiencies of factor V, VIII, and VWF have been reported in a patient with panhypopituitarism [7]. Factor IX deficiency has been reported in a patient with SS with hypothyroidism and hypocortisolism, which resolved after supplementation of thyroid hormone and cortisol [8]. Factor XI deficiency has not been reported in association with SS, and in our patient, the prolonged aPTT and factor XI deficiency persisted even after correction of hypothyroidism and hypocortisolism. This suggests that the factor XI deficiency was not due to hormone deficiencies but rather a chance association. However, it is possible that an underlying factor deficiency could have contributed to the occurrence of postpartum hemorrhage and the development of SS.

Factor XI deficiency is an autosomal recessive or autosomal dominant disease found predominantly in Ashkenazi Jews, although it is reported in non-Jewish populations with a lesser frequency. The prevalence of this disorder is estimated to be about 1 in 100,000 to 1 in 1 million. Clinically, the disease is highly variable, with a variable propensity to bleed excessively after trauma, particularly when the injury involves tissues rich in fibrinolytic activity such as the urinary tract, the nasopharynx, and the mouth. However, bleeding manifestations seem to correlate poorly with the level of plasma factor XI activity [9]. While post-traumatic hemorrhage can be significant, many patients with the severe form of the disorder (typically defined as a plasma factor XI activity ≤15% of normal) do not experience abnormal bleeding. Women with FXI deficiency (including heterozygotes) are at risk of menorrhagia and bleeding in relation to childbirth [10]. Approximately 20% of pregnant women with severe Factor XI deficiency experience excessive bleeding at the time of delivery in the absence of treatment. Our patient did not have any bleeding episodes in the past, except during the last delivery, which required a blood transfusion and may have contributed to the development of SS.

Our patient had secondary hypocortisolism, secondary hypothyroidism, and growth hormone deficiency. She had intermittent menstrual bleeding at an interval of about four to five months for the last nine years and had normal estrogen levels and an endometrial thickness of 8 mm on ultrasonography. This suggests preserved gonadotropin function, and she even had menstrual bleeding during her hospitalization. Her oligomenorrhea was likely due to secondary hypothyroidism and/or hypocortisolism. In SS, lactotrophs and somatotrophs are most severely involved, whereas gonadotrophs and corticotrophs are less involved, and pituitary dysfunction may develop slowly over several years after the episode of postpartum hemorrhage.

## Conclusions

We present the case of a woman with SS and congenital factor XI deficiency. This combination may have played a role in the onset of postpartum hemorrhage, eventually leading to panhypopituitarism. The simultaneous occurrence of both conditions in our patient is more likely a coincidental association than a direct causal relationship.
